# Evaluation of Bitumen Modification Using a Fast-Reacting SBS Polymer at a Low Modifier Percentage

**DOI:** 10.3390/ma16082942

**Published:** 2023-04-07

**Authors:** Juraj Šrámek, Matúš Kozel, Luboš Remek, Ján Mikolaj

**Affiliations:** Department of Construction Management, Faculty of Civil Engineering, University of Zilina, Univerzitna 8215/1, 01026 Zilina, Slovakia; juraj.sramek@uniza.sk (J.Š.); lubos.remek@uniza.sk (L.R.); jan.mikolaj@uniza.sk (J.M.)

**Keywords:** SBS polymer, bitumen modification, complex modulus, fatigue, asphalt mixture, cost evaluation, LCCA, life cycle evaluation

## Abstract

The study presented in this paper investigates the application of asphalt bitumen modification using a fast-reacting SBS polymer at a low modifier percentage. The hypothesis is that a fast-reacting styrene–butadiene–styrene (SBS) polymer that composes only 2% to 3% of the weight of the bitumen modification could extend the life of the pavement surfacing and pavement performance at relatively low input costs, increasing the net present value produced by the pavement during its life cycle. To confirm or refute this hypothesis, two types of road bitumens CA 35/50 and 50/70 were modified with low amounts of fast-reacting SBS polymer with the expectation of attaining properties similar to a 10/40–65 modified bitumen. For each type of unmodified bitumen, bitumen modification and comparative 10/40–65 modified bitumen, the following tests were conducted: needle penetration, softening point—ring and ball test method, and ductility test. The second part of the article focuses on a comparison of asphalt mixtures with different compositions of coarse-grain curves. For each mixture, complex modulus with varying temperatures and fatigue resistances are represented by the Wöhler diagram and compared. Based on in labo testing, the impact of the modification on pavement performance is evaluated. Life cycle changes for each type of modified and unmodified mixtures are quantified as road user costs, and attained benefits are compared with increased construction costs.

## 1. Introduction

The bitumen and bituminous binder are defined by current standards [1,2] as permanent, adhesive, hydrophobic, or water-resistant material obtained from crude oil. The properties of bitumen are highly influenced by its source and by the technological process of crude oil processing as described in case studies [3,4,5]. The most important parameters of bitumen for use in road construction are durability, easy processing ability, and visco-elastic behavior. In the line with the Shell Bitumen Handbook [6], the visco-elastic behavior of bitumen plays a large part in determining many aspects of road performance, particularly resistance to deformation and cracking, the two most common reasons for the structural failure of thicker pavements. Such parameters make bitumen one of the most used materials in pavement construction. Concerning the wide applicability, several approaches are known to improve the desirable bitumen properties impacting the final pavement layer mixture. The addition of a modifier is a common way to improve bitumen elasticity, rheological properties, the interaction of the binder in the resulting mixture, the ageing process, and overall resistance [7]. Most common modificants are plastomers and thermoplastic elastomers, including polyethylene (PE). The impact of PE on bitumen properties is highly dependent on its polymer structure. The addition of polymers reduces thermal susceptibility at higher temperatures, with a greater constancy of its properties. This is due to the fact that the bitumen becomes more elastic [8]. These improvements can be evaluated by the softening point test, penetration index test, and evaluation of rheological properties [9,10]. One of the main problems of PE modification is phase separation [9]. Other studies [10,11,12] found a significant impact of:

- PE dosage content ranges, with recommended modifier dosage from 3 to 6%,
- mixing temperature ranges, with recommended temperature from 160 to 185 °C,
- recommended mixing speed of 1300–5000 rpm,
- mixing duration ranging from 30 to 60 min.

Another problem is the impact of the storage stability of the PE modificant, concerning mainly the phase separation [13,14]. Storage stability was also studied by Vargas et al. [15], where mixtures containing PE are susceptible to phase separation after 72 h of storage at 160 °C.

Another approach is a modification with polypropylene (PP). This type of modification results in an improvement of resistance against moisture damage and increases the amount of the indirect tensile rate. PP modification is economically effective for PP content as little as 2% to 4%, i.e., very little modificant content [16].

Another type of modification is ethylene–vinyl acetate (EVA) and ethylene–butyl acrylate (EBA), with studies [17,18] indicating that such modification increases binder elastic responses and dynamic moduli at intermediate and high temperatures and reduces binder complex and stiffness moduli at low temperatures.

Low-density polyethylene (LDPE) and high-density of polyethylene (HDPE) can be used for bitumen modification. A study [19] has shown the significance of polymer particle size and storage stability. Based on [20], HDPE modification shows a more significant improvement of mechanical properties, however, at the cost of phase separation problems.

Previous research on styrene–butadiene–styrene (SBS) modification, concluded that bitumen mixture modified with SBS extracted from PET bottle/flakes and used in a concentration from 3 to 5% and a mixing speed of 5000 rpm for 2 h and temperatures between 170 and 180 °C has very high demands on storage conditions [21].

Bitumen can be modified with styrene–isoprene–styrene (SIS). A study [22] found that SIS combined with crumb rubber resulted in significant improvement in the softening point and temperature susceptibility of the modified mixture.

Finally, bitumen modified with styrene–ethylene/butylene–styrene (SEBS), in combination with montmorillonite nanoclay and modification ratio from 3 to 6%, improves the physical and rheological properties of produced bitumen mixture [23].

The practical application of bitumen modification, apart from increased production cost, are the high demands on storage conditions of produced bitumen and modificant mixture. This is due to phase separation. Appropriate storage conditions at batching plants demand a high energy input for heating. The current practice of SBS modified bitumen production is to use the entire amount of produced mixture. The unused bitumen and SBS mixture must be stored in heated storage tanks. If the SBS modified bitumen is stored without heating and mixing, serious phase separation appears, as is described in publications [24,25]. However, reference [26] shows that SBS modified bitumen can be stored for a short period. At SBS concentration of 3–6%, a decrease in the final properties of the stored mixture takes place in the first three days of storage, even if stored in heated tanks.

One possibility to apply SBS copolymer modification and reduce the impact of properties loss due to phase separation is the application of the modificant in the form of pellets directly into pre-heated aggregate during the mixing process prior to bitumen batching. The objective of the presented experiment is to ascertain whether this approach produces the expected properties improvement. The secondary objective is to ascertain whether a low percentage modification following this approach can be an effective way to produce improvement of the properties of the final bituminous mix. The premise is that the combination of these approaches could save a significant amount of production costs while providing benefits related to increased fatigue resistance of produced bound construction layers.

For the verification of this premise, the experiment was designed in the following way.

The first step was to compare bitumen without and with SBS modificant at varying concentration levels. This was carried out on road bitumen CA 50/70 and CA 35/50. Both were tested without modification and with 2, 2.5, 3, and 3.5% SBS modificant content. The tests were:

- penetration test,
- softening point test (ring and ball),
- ductility test.

The results were compared with properties of CA 10/40–65 with standard PMB modification from batching plant to ascertain the effectiveness of properties improvement compared with standard modification. The conclusion shows that, as expected, an increase in modificant correlates with properties improvement. For the following experiments, 3% SBS modification was chosen following the conclusions of the study [26], and 3% is also the least modificant concentration guaranteed by the producer to provide measurable improvements.

The following step entailed the preparation of three aggregate mixtures with different coarse lines. Such an approach is adopted concerning the impact of coarse-grain to the mixture parameters [27,28,29]. To create asphalt concrete samples, the three aggregates were used in combination with these asphalt binders:

- common road bitumen CA 50/70 without modification,
- modified road bitumen CA 50/70 with 3% SBS modification,
- modified road bitumen CA 10/40–65 with standard PMB modification from batching plant.

The tests performed on these samples were:

- complex modulus (E),
- fatigue measurement (ε_6_).

Complex modulus and fatigue parameters were used to ascertain the bearing capacity of the binder course layer. The bottom edge of the binder course layer must withstand repeated tensile stress produced by the traffic load. The bearing capacity is expressed as the loading cycles of the design axle load. These inputs are needed for adjustments of pavement performance models to evaluate the practical impact of modification in simulated traffic conditions.

## 2. Materials and Methods

**Figure 1:**
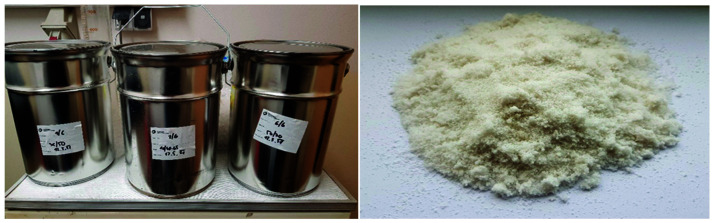
Materials for the first experiment of the interaction of straight run bitumen CA 35/50; 50/70 (**left**) and fast-reacting SBS polymer (**right**).

The materials for the experiment were obtained from common currently used materials in the middle Europe construction market. The bitumen producer is TOTAL, and the penetration and a softening point tests were performed on three different types of bitumen and one type of additional SBS modifier. Both materials used in the first series of tests are shown in Figure 1.

We split the measurements into the basic bitumen properties, and properties were measured on the different coarse-grain mixtures. In the results, we included the evaluation of residual service life based on fatigue characteristics of mixtures. The test results of comparison from in labo testing provide good insight into the performance of these paving materials. To translate these findings into measurable indicators impacting the decision-making process of a road administrator, i.e., their practical use, the following steps were taken to draw conclusions in the form of economic implications and technical considerations:

1. The in labo findings were translated into pavement performance models of pavements without and with low amounts of fast-reacting SBS polymer and standard polymer modification.
2. Computer simulation was performed in the Highway Design and Management tool (HDM-4). One lane road section was created and subjected to simulated climatic and traffic loading.
3. Pavement performance models created in Step 1 were used to model the life cycle of the road section, and life cycle analysis was performed. Life cycle analysis was performed for all three pavement types.
4. Life cycle cost analysis was performed for all three life cycles ascertaining construction cost and monetizing road user cost.
5. Conclusions were drawn to evaluate the economic viability of modification with low amounts of fast-reacting SBS polymer technology.

**Figure 2:**
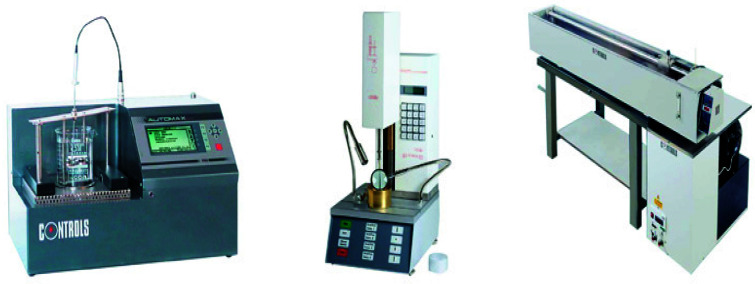
Testing devices from left to right—ring and ball, penetration needle, and ductility device.

The determination of basic properties of straight run bitumen and bitumen with fast reacting modification modifier in concentrations 2%, 2.5%, 3%, and 3.5% was carried out. The comparison value was obtained from the measurement of CA 10/40–65 bitumen. The basic test of modified bitumen was a penetration test at 25 °C according to EN 1426 [30], softening point test according to EN 1427 [31], and a ductility test according to EN 13398 [32]. For each type of test, two samples per dosage were performed. The test devices are shown in Figure 2. Each percentage amount of modifier was added into the preheated bitumen CA 35/50 and CA 50/70. A temperature from 160 to 170 °C was used. Each mixture was mixed in the rpm range from 700 to approx. 2000–2200 rpm for 45 min (started at 700 rpm for approx. 1min and then at 2200 rpm for 45 min).

### 2.1. Basic Properties of Bitumen and Modifier

The result of the first series of the in labo testing is shown in Table 1. As we can see, any additional modification of straight bitumen, as low as 2% of modification content, improves performance represented by a basic test of bitumen as softening point, ductility, and penetration. Compared with measured values of bitumen 10/40–65 whose penetration is 3.9, softening point is 65.8 °C, and elasticity ratio is 71%, the modification level is insufficient.

### 2.2. Properties of Asphalt Mixture

**Figure 3:**
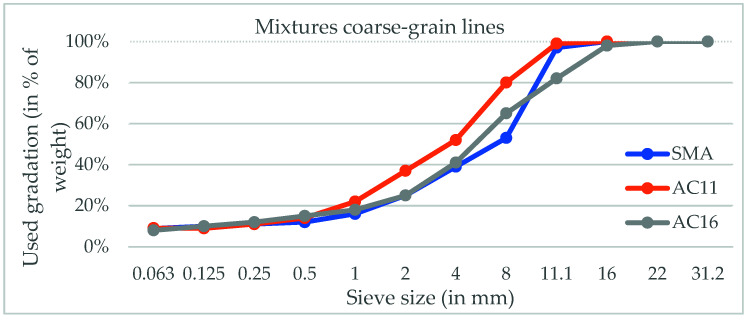
Used mixtures of coarse-grain lines, measured in the line with EN 12697-2.

In the second series of in labo tests, we carried out an experiment on batching plant, where different mixture types were prepared with the three commonly used coarse-grain lines, marked as SMA, AC11, and AC16. The shapes of each coarse-grain line estimated in the line with EN 12697-2 [33] are shown in Figure 3.

For each type of coarse-grain line used, comparison mixtures with other types of bitumen were prepared in the second step; thus, we prepared the next types of samples compacted by an asphalt slab roller compactor:

− binder course with max. grain 16 mm in the range to target a density of 2456 kg/m^3^:
  1. mixture with straight run bitumen CA 50/70
  2. mixture with modified bitumen 10/40–65
  3. mixture with additionally modified bitumen CA 50/70 with 3% fast-reacting polymer

− surface course with grain line corresponding to AC11, to target a density 2422 kg/m^3^:
  1. mixture with straight run bitumen CA 50/70
  2. mixture with modified bitumen 10/40–65
  3. mixture with additionally modified bitumen CA 50/70 with 3% fast-reacting polymer

− surface course layer with grain line corresponding to SMA, to target a density of 2391 kg/m^3^:
  1. mixture with modified bitumen 10/40–65
  2. mixture with additionally modified bitumen CA 50/70 with 3% fast-reacting polymer

For each such prepared sample, we measured rheology on the basis of the Boltzmann theory of linear viscoelastic materials, which better reflects the effects of repeated stress (fatigue), as described in detail in the literature [34,35,36]. Measurement of fatigue and complex modulus was carried out by EN 12697-24 [37,38].

## 3. Results

### 3.1. Rheology Properties of Different Mixtures

**Figure 4:**
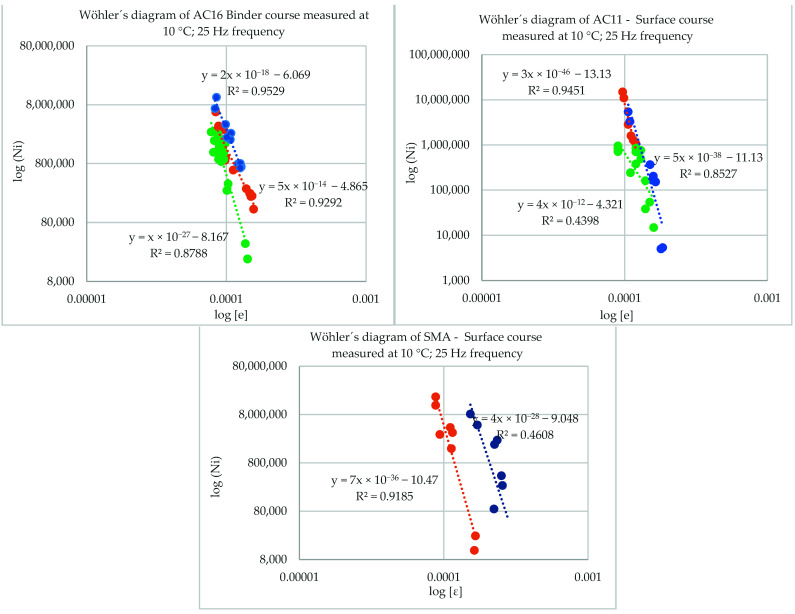
Wöhler’s diagram of evaluated mixtures, where CA 50/70 mixtures are marked in green, CA50/70 with 3% of SBS polymer mixtures are marked in orange, and 10/40–65 mixtures are marked in blue.

The result of the second series of the test is shown in Table 2 and Table 3. The results of fatigue measurement are described in Figure 4 for the binder course, for the surface course marked as AC11, and for the surface course marked as SMA.

The main factor in the assessment of fatigue resistance is precisely the expression of the fatigue characteristic ε_6_, which is defined for individual mixtures in terms of national regulations of individual countries. On the basis of measured values, we can estimate the service life of the pavement.

### 3.2. Service Life of Pavement Based on Different Fatigue Parameters of Asphalt Mixtures

The residual life expectancy calculation is based on the pavement structure design methodology, as described by Remek et al. [39,40,41]. Such a process is composed of rheological characteristics of pavement structure layers calculation and experimental measurement of fatigue parameter ε_6_ of asphalt mixtures of the pavement layers.

Fatigue parameter ε_6_ is calculated following Equation (1). The reliability of this calculation is dependent on the reliability at which the fatigue coefficients a and b are derived. These fatigue coefficients, *a* and *b*, are derived from experimental measurements of fatigue characteristics. They are parameters that represent the shape of Wöhler´s diagram (Figure 4), which expresses mixture resilience against repeated loading. The fatigue tests are carried out following European standards [37].

(1) $${log\;\varepsilon 0}_{j}=a_{j}+b\times logN$$

where

*ε0_j_* denotes maximum amplitude ordinate of proportional deformation during the test conditions at the beginning of the test,

*a* and *b* are fatigue parameters measured during the fatigue tests, presented as stress line coefficients in the range of *N*,

*N* represents the number of load repetitions.

Following the calculation of *ε_j_*, the calculation of maximum design axle load repetitions that the pavement can withstand can be determined from Equation (2):

(2) $$DAL=10^{6}\times {\left(\frac{\varepsilon_{6}}{\varepsilon_{j}}\right)}^{B}$$

where

*DAL* is number of design standard axle loads,

*ε_6_* denotes the average deformation derived from the fatigue curve after 10^6^ loading cycles in microstrain (µm/m),

*ε_j_* is the calculated relative deformation at the bottom of the critical bituminous bound sub-layer in the pavement construction (based on a multilayer system in homogenous half-space, calculation model); in our case, bottom edge of the binder course,

*B* is a fatigue characteristic specifying the falling gradient of the fatigue line, B = −1/b.

Concerning the topic of the article and comparison of mixtures, we simplify Equation (2) used in design methodology [42] for coefficient evaluating fluctuation of heavy axles on pavement, design speed of heavy vehicles, or other fatigue test reliability factors.

### 3.3. Service Life of Pavement Based on Different Fatigue Parameters of Asphalt Mixtures

Concerning the fatigue and the service life of the pavement in terms of the state of tension, the critical layer is the last bound layer in the direction from the road surface, in this model, the case is the binder course with an 80 mm thickness. The calculation of the service life, therefore, is performed on the same road structure (Table 3) but considers the different values of fatigue in the form of relative parameter ε6 and the complex modulus E of the binder course layer. The other parameters of the individual layers as well as the Poisson number for the binder course layer remained identical for the calculation.

If we calculate the residual life of selected pavement construction shown in Table 3 with different parameters of the binder course layer (fatigue parameters shown in Table 2), we obtain a total number of standard axle loads (DAL) such that pavement construction can carry on.

### 3.4. Pavement Performance and Life Cycle Analysis

Road deterioration ensues as a result of traffic loading and environmental/climatic effects. The paving material fatigue manifests itself as pavement deformation (IRI and RUT) and defects (cracks, potholes, ravelling, loss of skid resistance, etc.). The ability of the pavement structure and material to withstand these adverse effects is called pavement performance. The pavement performance model (PPM) is a mathematical description of the road deterioration process, and, as such, it is a crucial input for decision-making generally known as pavement management systems. The model used in this comparison is based on the ISOHDM study [43], which is utilized in the World Bank’s Highway Design and Management tool (HDM-4) [44].

**Figure 5:**
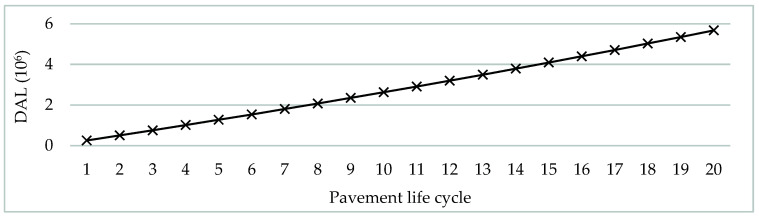
Number of design axle loads during the pavement’s life cycle.

**Figure 6:**
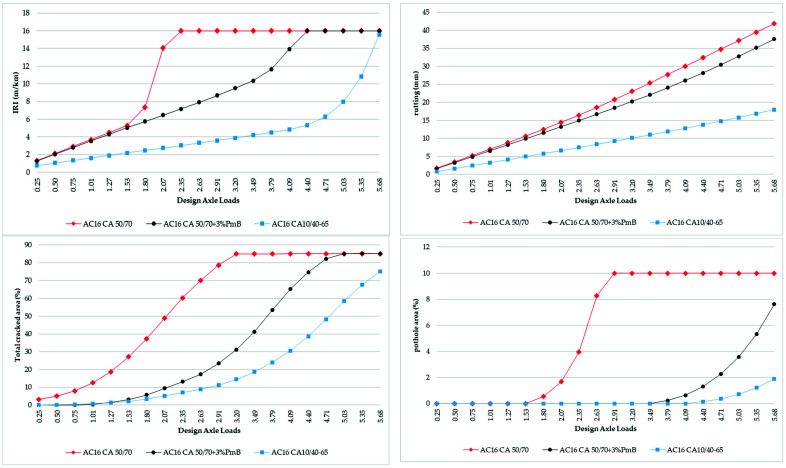
Pavement performance and life cycle analysis.

Three scenarios were evaluated following the in labo testing. The scenarios differ in one single parameter, and that is the binder course material. The base scenario is the binder course that is made from CA 50/70, and the following scenarios are CA 50/70 with 3% SBS binder course (modified with low amounts of fast-reacting SBS polymer and modified bitumen mixture) and CA 10/40–65 binder course. The differences in fatigue properties ascertained during in labo testing result in different values for an average annual adjusted structural number of the final pavement construction, proportionally to their respective in labo testing results. This denotes the higher bearing capacity that almost exactly matches the findings presented in Table 4. The pavement performance through the life cycle of pavement with surfacing made from these three paving materials was ascertained as a project analysis case study in the HDM-4 tool. The test evaluated 20 years of pavement’s life cycle for each pavement type. The climatic conditions (Table 5) remained constant during the analyzed period, and the initial traffic intensity rose by 1.5% annually (Table 6). During the 20-year life cycle period, the pavement accrued 5.68 × 10^6^ DAL (Figure 5). A pavement performance model [43] was applied for IRI, RUT, cracking, and pothole area. The pavement performance through the life cycle of pavement with binder course made from these three paving materials is shown in Figure 6.

### 3.5. Life Cycle Cost Analysis

#### 3.5.1. Capital Construction Costs

Capital construction costs represent the expenses incurred by the investor in the procurement phase and cover asphalt mixture producers’ and constructors’ direct overhead costs and profit. As all three pavement types differ only in the binder course, we can disregard the construction cost of all other pavement layers and base the evaluation only on the procurement cost of the binder course layer. In addition, since the construction technology is the same for all three scenarios, we can disregard the construction cost of the binder course and compare only the asphalt mixture producers’ cost. The market prices of asphalt mixtures AC16 CA 50/70 and AC16 CA 10/40–65 are readily available, as these are commonly used. The price of the asphalt mixture modified with fast-reacting SBS polymer was attained through inquiry surveys in several asphalt mixing plants. The capital costs in EUR per ton, cubic meter, square meter of 80 mm thick layer, and as total construction costs in the case study are shown in Table 7.

#### 3.5.2. Road User Costs

The road user costs monetized in the LCCA were:

- Travel time costs—passenger, crew, and cargo travel time. These are directly proportional to travel speed on the evaluated road section.
- Vehicle operating costs—fuel, oil, spare parts, maintenance, tire wear, interest, and depreciation costs of a vehicle. These are highly dependable on pavement serviceability in addition to travel speed.

**Figure 7:**
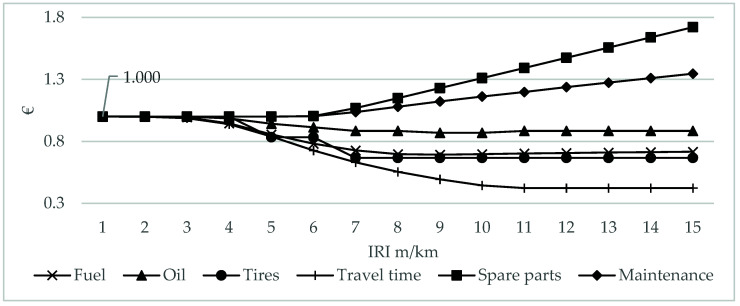
Road user cost related to IRI.

In the HDM-4 software v2.11, from the perspective of the relation between pavement serviceability and road user cost, longitudinal unevenness IRI is a composite parameter that has the main impact on road user cost and travel time. Figure 7 shows the general impact of pavement serviceability represented by IRI on road user cost. As IRI reaches 4 m/km and beyond, maintenance and spare parts costs increase. Degrading pavement serviceability impacts travel speed, which is the main driver of fuel, oil, and tire wear; a decrease in these vehicle operating costs, however, cannot outweigh the increase in travel time costs.

**Figure 8:**
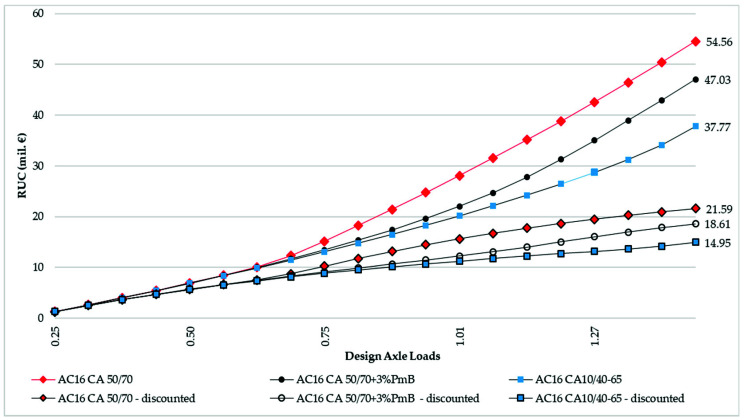
Cumulative road user cost: undiscounted and present value (discounted with 5% discount rate).

Figure 8 shows the cumulative road user costs, both undiscounted and discounted with a 5% discount rate, for all three scenarios. The sum of road user cost for the scenario with CA 50/70 unmodified binder course is EUR 54.65 mil., the present value of which is EUR 21.59 mil. The CA 50/70 with 3% SBS polymer binder course modified with low amounts of fast-reacting SBS polymer totals EUR 47.03 mil. of user cost, the present value of which is EUR 18.61 mil. In addition, the CA 10/40–65 binder course variant totals EUR 37.77 mil. user costs, the present value of which is EUR 14.61 mil.

## 4. Conclusions

As seen in Table 1, modification with low amounts of fast-reacting SBS polymer significantly improves the bitumen properties. As seen in Table 2 and Table 3 and Table 4, such improvements in basic asphalt properties do not have a significant increase in the fatigue resistance of the mixture. As seen in Table 4, modification with low amounts of fast-reacting SBS polymer can double the number of loading cycles the binder course can sustain. Full modification, however, can quadruple the number of loading cycles.

The price for a bitumen modification with low amounts of fast-reacting SBS polymer is currently only 1% higher than a standard modification.

The total sum of road user cost for a fast-reacting SBS polymer binder course scenario is EUR 22.37 mil., i.e., 10.90% higher than a standard PMB modification.

In economic terms, the previous three points mean that a EUR 1000 increase in capital costs for standard PMB modification (1% increase) will have an additional leverage of EUR 22.37 mil. of road user savings (10.9% increase) throughout a 20-year period. This means that the modification with low amounts of fast-reacting SBS polymer is not an economically effective option, although, realistically, the fast-reacting SBS polymer binder course scenario would end its life cycle as it reaches its critical pavement serviceability threshold, i.e., 7 years earlier, which would produce a mere 3.6% difference in road user savings, i.e., EUR 3.4 mil. This does not change the fact that full modification is still a more economically viable option. In addition, the binder course modified with full PMB modification would retain approximately one-third of its procurement asset value at this point.

A striking difference can be seen in the high procurement price of asphalt mixture modified with low amounts of fast-reacting SBS polymer compared with full PMB modification. This may be due to the fact that modification with low amounts of fast-reacting SBS polymer is not standardized, and this may inflate the initial mixture procurement price estimates. In addition, there are technical considerations that may make the fast-reacting SBS polymer modification technology worth considering in certain scenarios.

The value of the costs for the production of the mixture modified with SBS polymer (Table 7) did not reflect the reduction of costs associated with the storage of the mixture, as these are difficult costs to take into account and are directly dependent on the total amount of production of modified mixtures in individual periods and the setting of production–storage processes at individual asphalt mixture production plants. In addition, possible optimization of costs in the supply of modified mixtures was not taken into account. In the case of a reduction in the amount of stored mixture, we can assume a reduction in operating costs and, at the same time, a reduction in CO_2_ production per ton of produced asphalt mixture, as there is no energy consumption in the process of reheating/tempering and mixing the mixture during storage.

The limitation of the presented study is the translation of in labo testing by means of structural number changes to pavement performance. Structural number characterizes pavement strength, but its calculation method combines the surfacing course with the binder course. This may skew a little the results of pavement performance modeling, although, since this applies for all three scenarios, it should not change the proportionality of presented economic results.

Moreover, as stated in the economic implication discussion, the actual market procurement price of investigated experimental binder course modified with low amounts of fast-reacting SBS polymer cannot be estimated. It is safe to presume, however, that the price would be lower than that used in the comparison; thus, more favorable results can be expected if the production model of such a mixture is optimized. The following research steps should include FT-IR spectrometer measurement of selected SBS bitumen modificant for investigation of collaboration of bitumen and modificant. The ability of the material to withstand surfacing distress initiation and progression, as well as bearing capacity, should be explored. This would produce a more reliable pavement performance model and consequent road user cost calculation.

## Figures and Tables

**Table 1:** Basic properties of bitumen and modifiers.

Modifier Concentration (%)	CA 35/50			CA 50/70		
	Penetration (0.1 mm)	Softening Point (°C)	Ductility (%)	Penetration (0.1 mm)	Softening Point (°C)	Ductility (%)
0%	4.75	53.65	16%	6.74	49.65	12%
2%	5.34	57.05	48.75%	6.02	50.30	17%
2.50%	5.09	57.45	49.38%	5.76	50.50	23%
3.00%	4.83	57.55	51.02%	5.50	50.60	25%
3.50%	4.23	57.6	54.19%	4.79	50.40	29%

**Table 2:** Fatigue parameters of tested mixtures.

Type of Mixture	Fatigue Parameter			
	A_0_	B	b	ε_6_
AC16 CA 10/40–65	−17.7948	−6.0690	−0.1648	120.04
AC16 CA 50/70 with 3% SBS	−13.2967	−4.8649	−0.2056	108.02
AC16 CA 50/70	−26.9604	−8.1672	−0.1224	92.11
AC11 10/40–65	−37.3393	−11.1276	−0.0899	127.42
AC11 CA 50/70 with 3% SBS	−45.5806	−13.1257	−0.0762	117.56
AC11 CA 50/70	−11.4420	−4.3207	−0.2314	91.87
SMA 10/40–65	−27.4132	−9.0475	−0.1105	202.74
SMA CA 50/70 with 3% SBS	−35.1848	−10.4662	−0.0955	116.14

where ε_6_ is the strain level required for 1 million cycles fatigue life; b is the slope parameter of the fatigue line; B is the falling gradient of the fatigue line, B = −1/b; and A0 is an intersection point between the logarithmic function of strain measured at a sample failure (or upon reaching 50% decrease in the complex modulus) with a logarithmical function of loading cycles during fatigue testing.

**Table 3:** Pavement construction parameters.

Layer	Complex Modulus			Poisson Number	Layer Thickness
	CA 50/70	CA 50/70 with 3% SBS	CA 10/40–65		
Surface course	7578			0.33	40 mm
Binder course	10,504	693	7028	0.33	80 mm
Mechanically bound aggregate	587			0.30	180 mm
Gravel sub-base	366			0.30	200 mm
Sub-grade	100			0.35	-

**Table 4:** Total number of DAL.

Asphalt Type of Binder Course	CA 50/70	CA 50/70 with 3% SBS	CA 10/40–65
Total number of DAL	1.5 × 10^6^	3.4 × 10^6^	5.7 × 10^6^

**Table 5:** Climatic load.

Moisture classification	Semi-arid	Mean temperature	9.5 °C
Moisture index	−36	Avg. temperature range	20.2 °C
Duration of dry season	7.32 months	Days T > 32 °C	19.5 days
Mean monthly precipitation	54.5 mm	Freeze index	262

**Table 6:** Initial traffic load.

	Van	Medium Lorry	Passanger Car	Heavy Bus	Articulated Truck	Heavy Lorry	Total
AADT first year	149	43	4526	28	185	18	4949
20-year total	3450	986	104,655	657	4272	422	114,442

**Table 7:** Capital construction cost for binder course with the use of evaluated asphalt mixtures.

Binder Course Mixture	EUR/ton	EUR/m^3^	EUR/m^2^ (80 mm Binder Course)	1 km of 4.25 m Lane with 80 mm Binder Course
CA 50/70	94	263.20	21.06	89,488
CA 50/70 with 3% SBS polymer	116	332.80	26.62	113,152
CA 10/40–65	120	336.00	26.88	114,240

## Data Availability

Data available on request from the authors.
